# Vertical Farm Versus Glasshouse: Comparative Analysis of Growth, Antioxidants, and Volatile Profiles in Four Basil Varieties

**DOI:** 10.1002/pei3.70204

**Published:** 2026-09-01

**Authors:** Martina Pičmanová, Nick Bennett, Lidiya Babenko, Yogeswari Rajarathinam, Simon Pont, Ceri Austin, Tanveer Khan, Gordon McDougall, Derek Stewart

**Affiliations:** ^1^ Advanced Plant Growth Centre The James Hutton Institute Dundee UK; ^2^ Intelligent Growth Solutions Dundee UK

**Keywords:** aroma compounds, controlled‐environment agriculture, HS‐SPME‐GC‐MS, *Ocimum basilicum* L., phenolics

## Abstract

Global population growth and climate change are placing unprecedented pressure on food systems, threatening crop productivity and nutritional quality. Vertical farming, as a form of totally controlled‐environment agriculture, offers an innovative approach to optimizing plant growth and enhancing the quality of high‐value crops such as basil ( *Ocimum basilicum* L.). The aim of this study was to compare the growth performance, antioxidant properties, and volatile profiles of four basil varieties—Lemon Basil, Purple Basil, Sweet Basil, and Thai Basil—grown in a vertical farm (VF) and in a traditional semi‐controlled glasshouse (GH). In both environments, plants were cultivated in soilless systems with manual fertigation. Plant height, dry weight percentage, and the reflectance indices NDVI and Greenness Index were measured as indicators of growth performance; total phenolic content (TPC) and antioxidant capacity (FRAP) were analyzed to evaluate antioxidant status, and headspace solid‐phase microextraction gas chromatography‐mass spectrometry (HS‐SPME‐GC‐MS) was employed to characterize volatile compounds associated with the aroma of the crops. Depending on the basil variety, plants cultivated in VF generally exhibited enhanced growth compared to GH (up to 116% increase in plant height), increased antioxidant content (up to 88% higher TPC and up to 99% higher FRAP in VF), and greater accumulation of aromatic compounds (up to 120% larger summed peak area of the 50 most abundant volatile compounds in VF). These findings indicate that vertical farming can improve both growth and phytochemical quality of basil through precise environmental control, highlighting its potential for sustainable urban food production of high‐quality fresh produce.

## 1 Introduction

The world of the 21st century is facing unprecedented challenges caused by over‐population as well as by climatic change which is affecting crop production. Traditional agricultural systems are increasingly unable to meet food demands due to land degradation, water scarcity, and biodiversity loss (FAO 2017). Novel practices such as vertical farming have the capacity to transform food security by increasing productivity through continuous, year‐round crop production independent of seasonal and climatic constraints, by enhancing crop nutritional quality and by facilitating local food production and supply chain resilience (Kaiser et al. 2024). Vertical farms (VF) use Total Controlled‐Environment Agriculture (TCEA) for the cultivation of crops on vertically stacked trays. These systems integrate innovative LED lighting, climate control (including air temperature, CO_2_ concentration, relative humidity, and airflow) and sophisticated nutrient delivery technologies with controlled pH and electrical conductivity (EC) of the nutrient solution, thereby providing greater control over crop quality and productivity than traditional semi‐controlled glasshouses (GH). GH use natural sunlight as the primary light source, often supplemented with artificial light, and are typically single‐layer systems; environmental factors such as temperature and humidity are controlled, but they remain partially dependent on external climate conditions and seasonal variations (Vatistas et al. 2022). High‐tech vertical farms, with their artificial lighting and tightly controlled environment, have been proposed as alternatives or complements to glasshouses due to their potential to improve energy, water and land‐use efficiencies (Benke and Tomkins 2017; Graamans et al. 2018).

One of the flagship crops grown in VF is basil ( *Ocimum basilicum* L., Lamiaceae family), a popular herb with strongly aromatic leaves and health benefits, typically used in culinary, pharmaceutical, and personal care sectors (Azizah et al. 2023). Basil is well suited for both VF and GH systems as it is a fast‐growing, compact, and high‐value crop, well adapted to hydroponic and CEA systems (Sipos et al. 2021). Although open field and GH production of basil far exceeds vertical farming in total volume due to their widespread and well‐established operation, vertical farming contributes a growing share as it is expanding rapidly with urban agriculture investments (Karpe et al. 2025).

Although basil has high nutritional content, including vitamins, carotenoids, minerals and health‐beneficial antioxidant phenolic compounds such as rosmarinic, chicoric, gallic, *p*‐hydroxybenzoic, ferulic, and caffeic acids (Flanigan and Niemeyer 2014; Kopsell et al. 2005; Rana et al. 2022; Sgherri et al. 2010), it is particularly valued for its high content of essential oils consisting of a wide range of volatile compounds, including monoterpenoids, sesquiterpenes and phenylpropanoids, which are responsible for its typical flavor and aroma (Lee et al. 2005). However, the production of these volatile compounds can be influenced by environmental conditions, including light (intensity, spectrum, and photoperiod), temperature and nutrient availability (Aghamirzaei et al. 2024; Carvalho et al. 2016; Walters and Lopez 2022). Moreover, plants often exhibit genotype‐by‐environment interactions, resulting in species‐ and varieties‐specific responses, including variation in growth and quality traits, such as volatile profiles in aromatic herbs (Chutimanukul et al. 2022; Jiang et al. 2024; Kelly et al. 2025). Within TCEA systems, environmental conditions can be strategically optimized to tailor plant growth and aroma composition to consumer preferences and market demands (Kelly et al. 2025).

While previous studies have investigated basil performance under different controlled‐environment cultivation systems, including a direct comparison of growth chamber and GH production (Carvalho et al. 2016), comparative studies involving multiple basil varieties grown under VF and GH conditions are scarce.

This preliminary study aimed to investigate how the controlled and semi‐controlled environments of VF and GH, respectively, affect the growth parameters, antioxidant content, and volatile profiles of four basil varieties—Lemon Basil, Purple Basil, Sweet Basil, and Thai Basil. We hypothesized that the greater level of environmental control in VF would result in altered growth characteristics as well as different contents and compositions of volatiles and antioxidants compared with GH, with responses differing among varieties. As a systems‐level comparison of real‐world production environments, the study provides insight into the integrated effects of multiple environmental factors on basil growth and quality. The potential of vertical farming for the production of health‐promoting aromatic plants is discussed.

## 2 Material and Methods

### 2.1 Plant Material and Cultivation

The experiment was conducted in April–May 2024 in the state‐of‐the‐art vertical farm at the Intelligent Growth Solutions Ltd. Crop Research Centre and in a glasshouse at the James Hutton Institute (Invergowrie, Scotland). The seeds of four basil varieties, namely Lemon Basil ( *Ocimum basilicum* var. *citriodora*), Purple Basil ( *Ocimum basilicum* “Red Shiraz”), Sweet Basil ( *Ocimum basilicum* “Genovese”), and Thai Basil ( *Ocimum basilicum* var. *thyrsiflora*) were purchased from CN Seeds (Ely, United Kingdom). The seeds were sown using a Mosa Drum Seeder sowing machine (Mechanical Botanical, Chiddingfold, United Kingdom) into 40‐cell growth trays (310 × 530 mm, HerkuPlast Kubern, Germany), with 1–2 seeds sown per cell, filled with a coconut coir (Jiffy Indoor farming coir, Netherlands) and vermiculite (Plant!t, United Kingdom) substrate medium at a 75:25 (v/v) ratio. The growth trays were incubated in a dark germination chamber (25°C, 90% RH) for 3 days and then placed on liners in the large growth tray with lights (GTL) in the VF growth tower and in the GH. Each growth tray contained on average 56 germinated plants and represented one biological replicate. Plants were harvested 25 days after sowing.

### 2.2 Environmental Conditions

In VF, narrow‐band LED lights (Osram, Austria) were arranged horizontally 300 mm above the tray base in the GTLs and emitted blue light [peak emission at 451 nm, full width at half maximum (FWHM) 18 nm], green light (peak emission at 521 nm, FWHM 30 nm) and hyper‐red light (peak emission at 660 nm, FWHM 25 nm) at changing ratios, with the photosynthetic photon flux density (PPFD) of 261–326 μmol m^−2^ s^−1^, the daily light integral (DLI) of 16.9–21.1 mol m^−2^ day^−1^ and the photoperiod of 18 h day^−1^. The five‐phase light recipe is described in detail in Table 1. All trays were positioned within the predefined area of uniformity and were therefore assumed to have experienced equivalent light intensity and spectral composition across varieties and positions. Uniformity was determined using the central 75% of GTL area, with minimum‐to‐mean light uniformity ratio ranging from 0.95 to 0.96, and the minimum‐to‐maximum ratio ranging from 0.91 to 0.94, confirming high spatial consistency of irradiance. In GH, the plants were grown under natural light supplemented with white LEDs (Vertically Urban, United Kingdom) with an off threshold of 150 W m^−2^, 18‐h photoperiod, and an estimated average PPFD of 300 μmol m^−2^ s^−1^. The total DLI (PAR fraction of natural sunlight with supplemental lighting) in GH was estimated from the hourly meteorological data (The James Hutton Institute Weather Station) between 22 and 40 mol m^−2^ day^−1^. The temperature was set at 24°C in both systems but in GH fluctuated significantly depending on the weather conditions and the time of the day, with temperatures routinely exceeding 35°C on sunny days while remaining around 24°C at night. In VF, RH was maintained at 70% and CO_2_ concentration at 800 ppm; in GH, RH varied with temperature fluctuations, averaging approximately 38%, while CO_2_ concentration was estimated to be close to ambient atmospheric levels (approximately 420 ppm). The same standard nutrient solution (NPK molar ratio 5:1:4, EC 2.0, pH 5.8) was used for manual fertigation in both VF and GH systems. The fertigation regime was kept identical in both systems: 7 L of nutrient solution were applied to each liner twice a week and either drained after 1 h during the first 2 weeks or allowed to remain in the liner in the final week; in GH, one additional irrigation event with pure water only was required to prevent wilting during a hot, sunny day.

**TABLE 1:** A five‐phase light recipe used to grow four basil varieties in VF.

Phase	Duration (days)	PPFD (μmol m^−2^ s^−1^)	DLI (mol m^−2^ day^−1^)	Red:Green	Red:Blue	Blue:Green
1	7	261	16.91	21.63	2.16	10
2	3	294	19.05	16.83	2.53	6.67
3	4	298	19.31	12.63	2.53	5
4	3	320	20.74	9.43	2.71	3.48
5	5	326	21.12	7.48	2.71	2.76

Abbreviations: DLI, daily light integral; PPFD, photosynthetic photon flux density.

### 2.3 Sample Preparation

Plant height was recorded and reflectance spectra for Normalized Difference Vegetation Index [NDVI (R_NIR_ − R_RED_)/(R_NIR_ + R_RED_)] (Rouse et al. 1974) and Greenness Index (GI; R_554_/R_677_) were measured using PolyPen RP410 (UV–VIS; Photon Systems Instruments, Czech Republic) at the end of the growth period in five plants randomly selected from different positions within each growth tray (excluding plants from the longer‐edge rows, which were in contact with plants from neighboring trays). After collecting fresh samples for SPME‐GC‐MS analysis, the plants from each growth tray were harvested, pooled and thoroughly mixed to form a single composite sample (excluding plants from the longer‐edge rows, as described above). A representative subsample was subsequently weighed, snap frozen in liquid nitrogen and freeze‐dried using Christ Gamma 1–16 LSC freeze dryer (Osterode am Harz, Germany) for 3 days. The dry matter was established to determine the percentage of dry weight, and the plant material was homogenized using Retch ZM 200 ultracentrifugal mill with sieve size 0.5 mm (Haan, Germany). Samples were kept in 50 mL‐falcon tubes at −20°C until required for analysis.

### 2.4 HS‐SPME‐GC‐MS Analysis

One leaf disc (1.4 cm in diameter, area of 1.54 cm^2^) was excised using a cork borer from the same lamina region of the first fully unfolded leaf (from the apex) of six randomly selected individual plants on a growth tray (excluding plants from the longer‐edge rows, as described above). These six leaf discs were inserted into a headspace vial, capped and stored at −20°C until analysis. The samples were defrosted and equilibrated on the GC tray at room temperature for 16 h and directly analyzed using Thermo Scientific Trace GC Ultra gas chromatograph coupled to a DSQII quadrupole mass spectrometer (Hemel Hempstead, United Kingdom), and equipped with a PAL autosampler (CTC Analytics, Switzerland). Polydimethylsiloxane/divinylbenzene (PDMS/DVB) SPME fiber (75 μm coating with a 23‐gauge protective sheath; Supelco, United Kingdom) was conditioned under N_2_ flow at 250°C for 30 min in the autosampler conditioning port and placed onto the fiber holder before analysis. The headspace vials with samples were incubated at 35°C for 2 min and the fiber was subsequently exposed to each sample headspace volatiles for 2 min at the same temperature. The volatiles extracted on the fiber were then desorbed onto the GC‐inlet at 250°C for 2 min. The GC separation was performed on a DB‐1701 capillary column with helium as a carrier gas at a constant flow rate of 1.5 mL/min and the following gradient: 0–2 min at 40°C, ramp at 10°C/min until 240°C and hold at 240°C for 10 min, with a split ratio 1:50. Mass spectra were acquired at 6 spectra s^−1^ over the mass range 35–400 Da, under electron impact (EI) ionization conditions (positive mode) at 70 eV, with a source temperature of 200°C. Data were acquired and subsequently analyzed using the Xcalibur software package version 2.2 (Thermo Scientific, United Kingdom). Detected volatile compounds were annotated based on their spectra, NIST MS Search 2.4 mass spectral database match and comparisons with authentic standards, where available. Relative abundance of volatiles was calculated from integrated peak areas and normalized to leaf area (cm^2^).

### 2.5 Antioxidants

Total phenolic content (TPC) and antioxidant capacity (Ferric reducing antioxidant power assay; FRAP) were determined as described previously by Bucky et al. (2024). Briefly, to extract antioxidants, 1 mL of 80% methanol was added to 20 mg of pulverized samples, the suspensions were then vortexed for 30 s, sonicated in an ultrasonic bath with ice for 15 min, and agitated on an orbital shaker for 30 min. The samples were then centrifuged for 10 min at 15,000 rpm, and the supernatants stored at −80°C. For TPC analysis, 250 μL Folin–Ciocalteu reagent and 500 μL of saturated sodium carbonate were added to 250 μL of 10‐times diluted samples, and the mixes incubated for 1 h at room temperature in three technical replications. The absorbance was measured spectrophotometrically at 750 nm, and a gallic acid standard curve (0.01–0.1 mg mL^−1^, *r* ^2^ = 0.999) was used for quantification. FRAP was performed by adding 900 μL of FRAP reagent (300 mM sodium acetate buffer, pH 3.6; 10 mM 2,4,6‐Tris(2‐pyridyl)‐s‐triazine in 40 mM HCl; and 20 mM FeCl₃·6H_2_O mixed in a 10:1:1 ratio) to 100 μL of 20‐times diluted methanolic extracts in three technical replications, and the absorbance measured at 593 nm exactly 4 min after reagent addition. Antioxidant capacity was quantified using a FeSO_4_ standard curve (0.1–1 mM; *r* ^2^ = 0.999). Results were expressed in g of gallic acid equivalent (GAE) per 100 g of sample on dry weight basis for TPC and as mmol Fe^2+^ per 100 g of sample on dry weight basis for FRAP.

### 2.6 Experimental Design and Data Analysis

The experiment followed a split‐plot design. Environment (VF or GH) was the whole‐plot factor, with three independent liners per environment serving as replicate blocks. Each liner contained four growth trays, with each growth tray randomly assigned to one of the four varieties (the subplot factor). Statistical analyses were conducted using liners as the whole‐plot experimental units and growth trays as the subplot experimental units. Data were analyzed in Genstat (22nd Edition, VSNi, United Kingdom) using split‐plot ANOVA, with environment and variety treated as fixed effects and liner as the blocking factor nested within environment. Liner provided the whole‐plot error term for testing the effect of environment, whereas the residual error was used to test the effects of variety and the environment × variety interaction. Mean comparisons were performed using Fisher's protected least significant difference (LSD) test at *p* < 0.05. As the environmental treatments were applied at the facility level, inference regarding the environment effect is limited to the specific VF and GH evaluated in this study. A hierarchical clustering heatmap was generated using MetaboAnalyst 6.0 (Pang et al. 2024).

## 3 Results and Discussion

**FIGURE 1:**
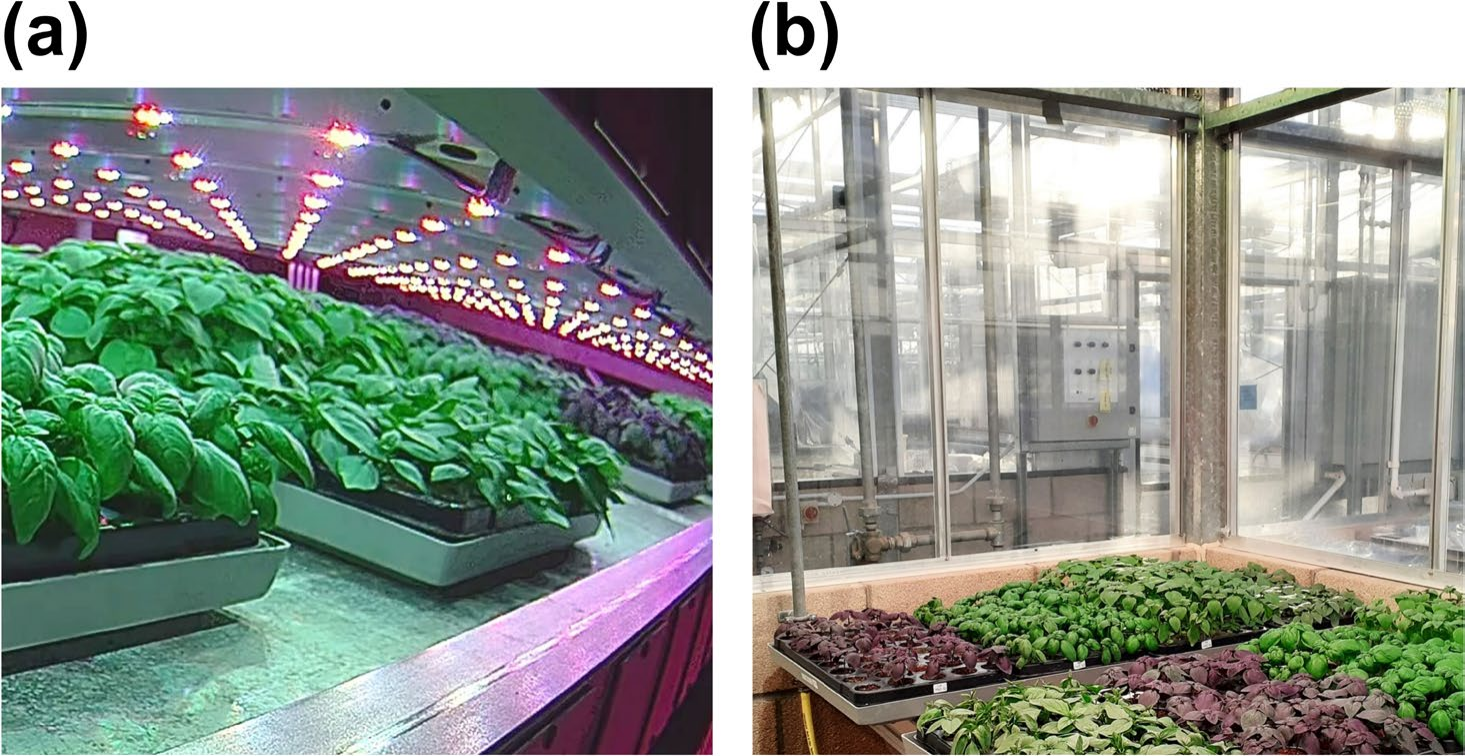
Growth environments used for cultivation of four basil varieties in this study. (a) Representative photograph of basil plants grown in VF (totally controlled environment), captured by the facility's internal monitoring system; (b) Representative photograph of basil plants grown in GH (semi‐controlled environment).

**FIGURE 2:**
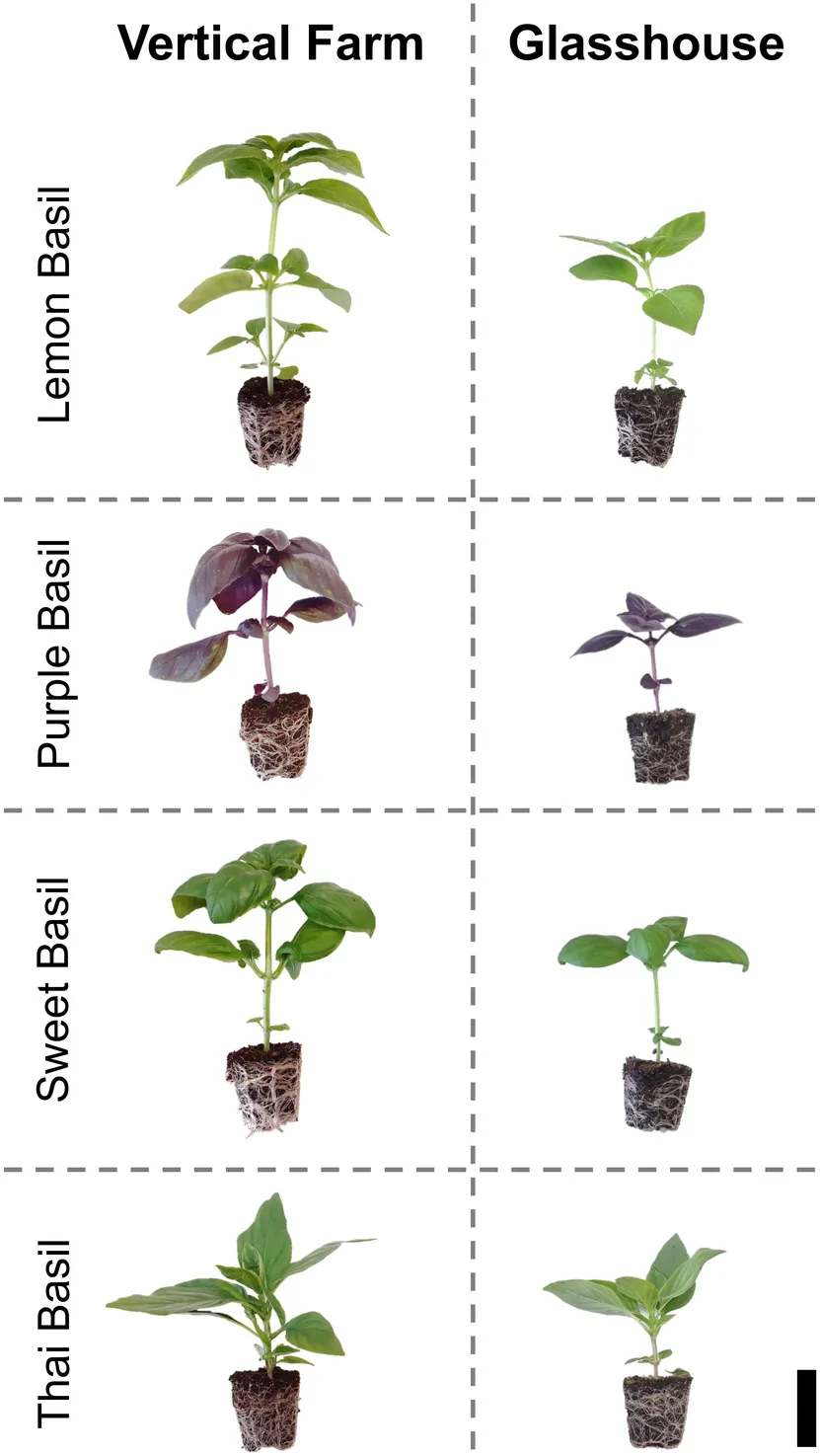
Representative plants of four basil varieties grown in VF and GH. Plants were photographed at harvest 25 days after sowing. Scale bar = 5 cm.

**FIGURE 3:**
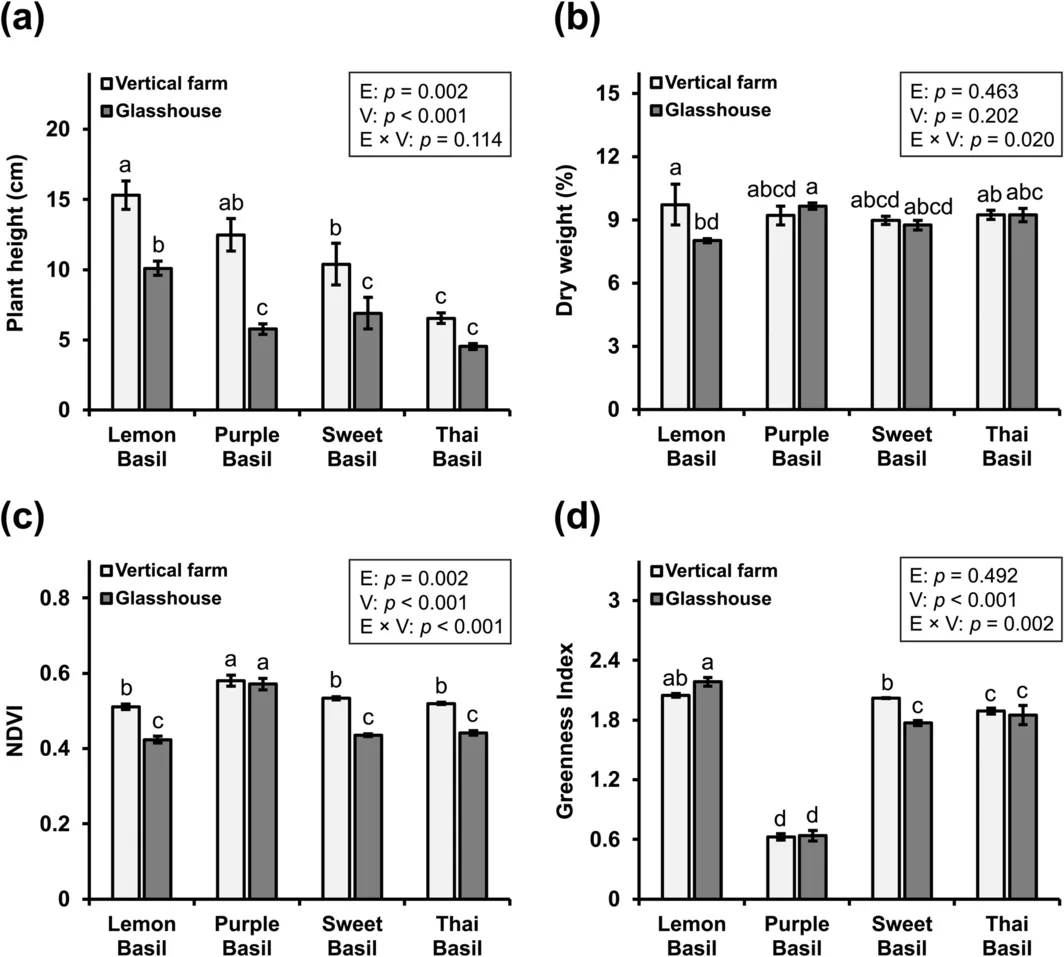
Growth parameters in four basil varieties grown in VF and GH. (a) Plant height, (b) Dry weight percentage, (c) Normalized Difference Vegetation Index (NDVI) and (d) Greenness Index. Bars represent mean values ± SE (*n* = 3). Different letters indicate significant differences among all Environment (E) × Variety (V) treatment combinations (split‐plot ANOVA, Fisher's protected LSD, *p* < 0.05).

**FIGURE 4:**
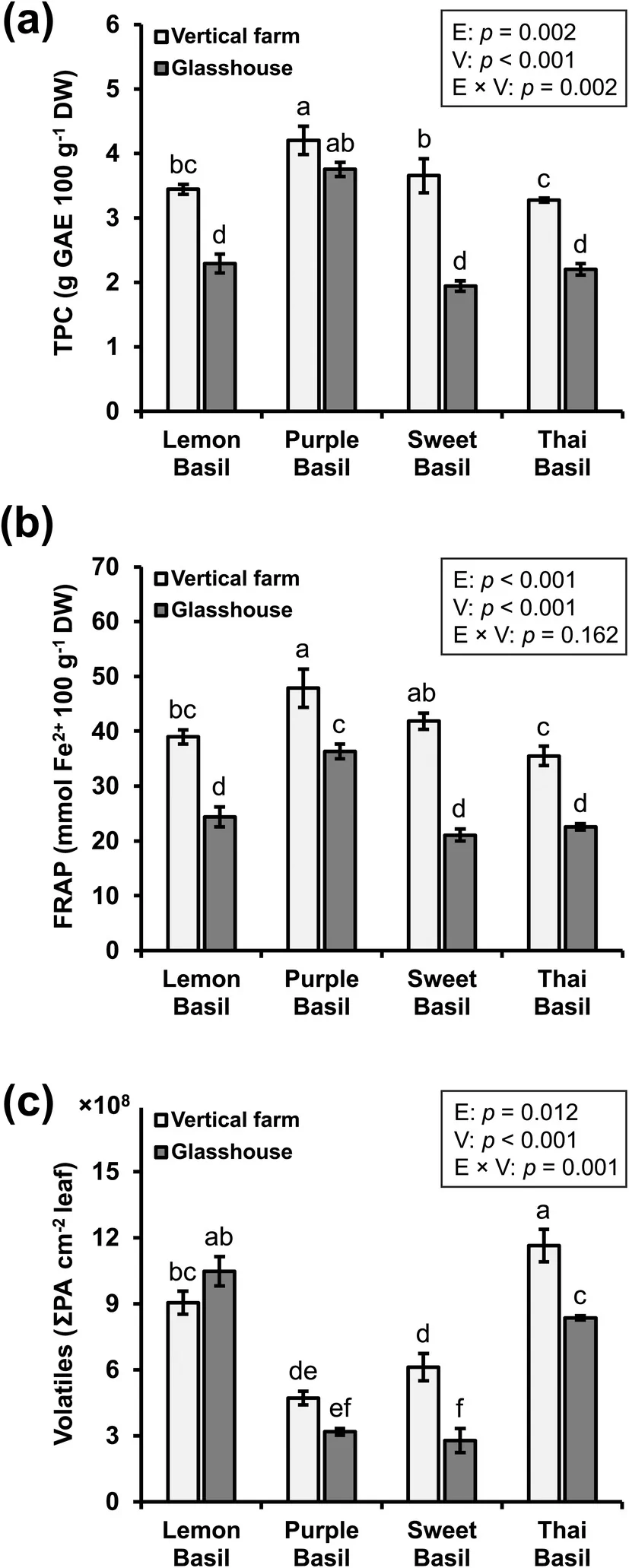
Comparison of antioxidants and volatiles in four basil varieties grown in VF and GH. (a) Total phenolic content (TPC); (b) FRAP antioxidant capacity; (c) Sum of 50 major volatile peak areas (ΣPA) per cm^2^ of leaf. Bars represent mean values ± SE (*n* = 3). Different letters indicate significant differences among all Environment (E) × Variety (V) treatment combinations (split‐plot ANOVA, Fisher's protected LSD, *p* < 0.05).

**FIGURE 5:**
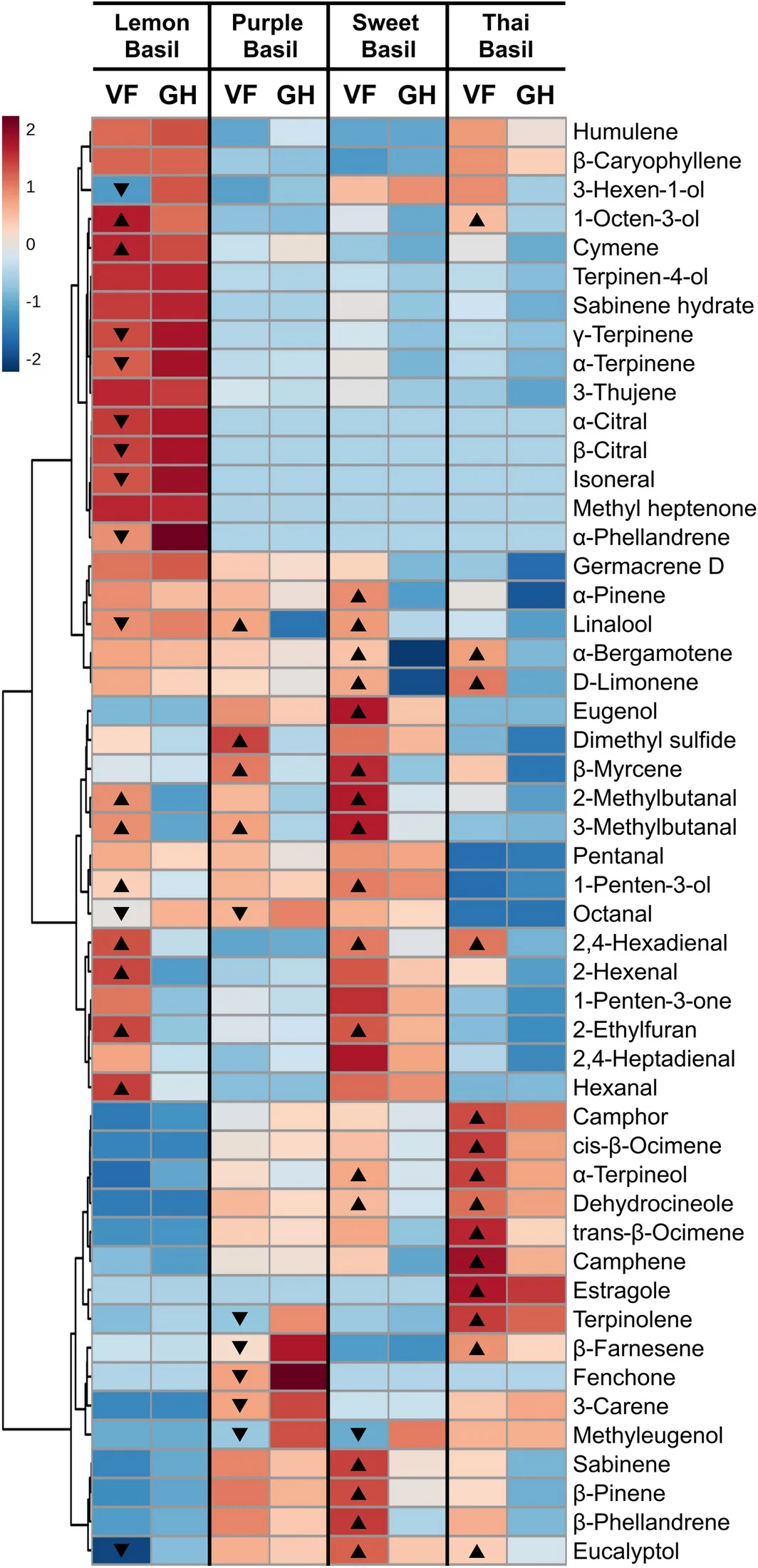
Heatmap showing relative peak areas per cm^2^ of leaf of 50 annotated major basil volatiles, as generated by MetaboAnalyst 6.0; values are log_10_‐transformed and auto‐scaled by individual volatile compounds (rows) for visualization purposes, with red indicating higher and blue indicating lower relative peak areas (color intensities to be compared across samples only); hierarchical clustering was performed using Euclidean distance and Ward method; ▲ marks significant increase and ▼ marks significant decrease of volatile intensities in pairwise comparisons VF vs. GH for each compound and variety (split‐plot ANOVA, Fisher's protected LSD, *p* < 0.05). Dehydrocineole, 2,3‐Dehydro‐1,8‐cineole; Methyl heptanone, 6‐Methyl‐5‐hepten‐2‐one.

We investigated how controlled versus semi‐controlled environments modulate both growth and quality attributes in four basil varieties, when cultivated hydroponically using an identical nutrient solution (Figure 1). The results indicate that vertical farming generally offers a more favorable environment for basil growth and for its phytochemical content, with the effect depending on the variety (Figures 2, 3, 4, 5, Tables S1–S3). Plants grown in VF were overall larger and taller than plants grown in GH (Figures 2 and 3a), although plant height was not significantly different in Thai Basil (Figure 3a). Plant height ranged from 4.5 cm (GH‐grown Thai Basil) to 15.3 cm (VF‐grown Lemon Basil), with plants in VF exhibiting height increases of 44%–116% relative to GH‐grown plants (Figure 3a). VF‐grown basil plants appeared visibly larger, with a greater number of expanded leaves and a more developed root system, consistent with greater overall growth vigor (Figure 2). However, dry weight % was consistent across varieties and environments (9.3% on average), with the only exception being GH‐grown Lemon Basil which had a significantly lower dry mass percentage (8%) and therefore a higher water content (Figure 3b). These findings suggest that VF conditions favored more efficient growth and resource allocation without inducing changes in biomass/water content ratio. The enhanced growth observed in VF likely reflects the more stable and optimized cultivation conditions compared to GH, including consistent diurnal temperature and relative humidity, elevated CO_2_ concentrations, and precisely controlled light quality and quantity, all of which are known to promote photosynthesis, carbon assimilation and biomass accumulation (Farhangi et al. 2023).

Two selected reflectance indices, Normalized Difference Vegetation Index (NDVI) and Greenness Index (GI) were calculated as indicators of plant health, reflecting chlorophyll content and leaf structural properties that are associated with the plant's capacity to intercept photosynthetically active radiation and channel it into photosynthesis. In our measurements, NDVI was significantly higher in VF across varieties, except for Purple Basil, for which values were similar under both environments (Figure 3c). The higher NDVI values observed in VF therefore suggest that the optimized and controlled conditions promoted a more favorable physiological status and photosynthetically active leaf tissue compared with GH‐grown plants. Conversely, GI indicated no strong environmental effect except for significantly lower GI in Sweet Basil grown under GH conditions, potentially due to the stress caused by diurnal temperature and light swings (Figure 3d). Despite greater plant vigor in VF‐grown plants suggested by NDVI, GI indicates that leaf chlorophyll content was largely maintained across environments. The significantly lower GI observed in Purple Basil compared with the green varieties is likely attributable to the high accumulation of anthocyanins (Flanigan and Niemeyer 2014), which alter leaf reflectance in the visible spectral bands used for GI calculation; anthocyanins mask the green coloration of chlorophyll, thereby reducing the apparent greenness signal independently of chlorophyll concentration (Figure 3d; Merzlyak et al. 2008).

The total phenolic content (TPC) and the related FRAP antioxidant capacity showed very similar patterns, indicating that phenolics make a major contribution to basil antioxidant properties. Both TPC and FRAP were significantly higher (49%–88% and 32%–99% increase, respectively) in all the basil varieties grown in VF, with the exception of Purple Basil, for which TPC did not differ significantly under the two environments (Figure 4a,b). Purple Basil had the highest levels of phenolics as well as the highest antioxidant capacity, most likely due to its high content of the purple pigments anthocyanins which are well known antioxidants with neuroprotective, cardioprotective and anti‐inflammatory benefits (Flanigan and Niemeyer 2014; Liu et al. 2025). However, the antioxidant capacity of VF‐grown Sweet Basil (a green variety with no visible anthocyanins) was significantly higher than that of GH‐grown Purple Basil, suggesting that components other than purple pigments were driving the high antioxidant capacity, possibly other phenolics such as rosmarinic, *p*‐hydroxybenzoic, gallic, and other phenolic acids (Sgherri et al. 2010; Ryu et al. 2026). The stimulatory effect of the VF environment on the production of specialized metabolites with antioxidant properties may be associated with the more eliciting controlled light of the narrow‐band LEDs compared to the spectrally broader, diffused and glass‐filtered solar radiation in GH (Castilla 2013). In particular, high blue light is known to activate cryptochrome‐mediated signaling, resulting in enhanced phenylpropanoid metabolism and higher phenolic accumulation (Chen et al. 2026). Light spectra with high blue:red ratios have been previously shown to affect positively both TPC and antioxidant capacity in basil and other crops (Bucky et al. 2024; Chutimanukul et al. 2022; Jokic et al. 2025).

The relative intensities and profiles of volatile compounds differed dramatically among the four basil varieties and environments (Table 2, Figures 4c and 5, Tables S2 and S3). The sum of volatile peak areas per cm^2^ of leaf (ΣPA, 50 major volatiles) was highest in Thai and Lemon Basil, whereas Purple and Sweet Basil exhibited comparable values that were significantly lower than those observed in the other two varieties. ΣPA was significantly lower under the GH conditions in Sweet Basil (by 55%) and in Thai Basil (by 28%) compared to VF. A similar effect, albeit not statistically significant, was observed in Purple Basil. Volatiles accumulation was found in GH‐grown Lemon Basil, but the difference between GH and VF was not statistically significant (Figure 4c). The profile of Lemon Basil was dominated by citral‐related volatile compounds (with citrus‐like aroma) and other oxygenated monoterpenes (especially linalool with lavender‐like aroma), while the profile of Thai Basil contained a large proportion of phenylpropanoids (especially estragole with anise‐like aroma), and Sweet/Purple Basil profiles were driven by the monoterpenoids eucalyptol (eucalyptus‐like aroma) and linalool (Table 2, Figure 5). Notably, the terpenoids α‐/β‐citral and linalool were significantly lower in Lemon Basil grown in VF, whilst in Sweet Basil the terpenoids eucalyptol, linalool and several monoterpenes, such as D‐limonene, α‐ and β‐pinene and β‐myrcene, were significantly higher in VF compared to GH (Figure 5). In Purple and Sweet Basil, the phenylpropanoid eugenol and its methylated derivative methyleugenol were affected by the two environments in an opposite fashion: eugenol levels were higher in VF whilst methyleugenol was significantly higher in GH, suggesting potentially stress‐induced upregulation of eugenol *O*‐methyltransferase activity in GH‐grown plants (Renu et al. 2014). In Thai Basil, many of the major volatiles including estragole and eucalyptol were significantly higher under VF conditions. Given that basil varieties differ in their dominant volatile biosynthetic pathways, environmental conditions play a key role in shaping volatile accumulation patterns (Escobar‐Bravo et al. 2023). Accordingly, VF through optimized lighting and controlled growth conditions may modulate volatile profiles in genotype‐dependent ways, whereas the fluctuating light and temperature typical in GH cultivation possibly reflects mild abiotic stress that modifies specific volatile compounds via stress‐related secondary metabolism (Branca et al. 2024; Carvalho et al. 2016). Together, these environment‐dependent modulations may have important consequences for the aromatic and flavor qualities of basil (Hammock et al. 2021; Kelly et al. 2025).

**TABLE 2:** Percentage contribution of dominant volatile compounds and volatile classes to the total volatile peak area (ΣPA) per cm^2^ of leaf in four basil varieties grown in VF and GH.

Volatile compound/class	% ΣPA cm^−2^ leaf							
	Lemon basil		Purple basil		Sweet basil		Thai basil	
	VF	GH	VF	GH	VF	GH	VF	GH
α‐/β‐Citral (OM)	37.3	42.2	n.d.	n.d.	n.d.	n.d.	n.d.	n.d.
Eucalyptol (OM)	5.6	7.9	31.6	42.2	30.6	49.5	10.6	12.6
Linalool (OM)	28.3	30.7	41.0	2.3	36.3	15.2	2.8	0.7
Estragole (PP)	n.d.	n.d.	n.d.	n.d.	n.d.	n.d.	60.8	71.4
**Subtotal**	**71.1**	**80.9**	**72.6**	**44.5**	**66.9**	**64.7**	**74.2**	**84.7**
Aliphatic aldehydes/ketones	5.4	2.4	5.8	9.1	7.6	12.9	2.7	2.8
Aliphatic alcohols	5.8	2.5	0.7	0.9	1.1	1.0	1.2	0.4
Monoterpenes	9.8	6.6	16.2	19.4	18.9	10.5	13.5	6.4
Oxygenated monoterpenes	77.1	87.0	75.2	51.8	69.6	67.7	19.8	18.3
Sesquiterpenes	1.7	1.3	1.3	1.7	1.0	0.2	0.9	0.3
Phenylpropanoids	n.d.	n.d.	0.4	16.8	1.5	7.1	61.8	71.7
Sulfides/Furans	0.2	0.1	0.4	0.3	0.4	0.6	0.1	0.1
**Classes total**	**100.0**	**100.0**	**100.0**	**100.0**	**100.0**	**100.0**	**100.0**	**100.0**

*Note:* ΣPA cm^−2^ leaf is the sum of peak areas per cm^2^ of leaf of the 50 major volatile compounds. More details can be found in Tables S2 and S3.

Abbreviations: n.d., not detected; OM, oxygenated monoterpene; PP, phenylpropanoid.

This study indicates that VF may offer significant advantages over GH cultivation for the production of different basil varieties in terms of plant growth, antioxidant and volatile compound content. Depending on the variety, basil plants grown in the vertical farm were generally healthier, more aromatic, and grew faster, allowing an estimated four to five additional harvests per year. These observations might be attributed primarily to the environmental stability, optimized light conditions, and CO_2_ enrichment in VF, in contrast to the suboptimal conditions characterized by variable light, temperature, and humidity fluctuations and ambient CO_2_ levels in GH.

Future research should focus on elucidating the mechanisms underlying antioxidant and volatile compound biosynthesis under different environmental conditions, alongside the evaluation of the potential of vertical farming systems for the cultivation of other aromatic crops or alternative targets such as pharmaceutical crops. The controlled conditions inherent to vertical farming enable precise optimization of growth parameters, as well as the targeted modulation of metabolic profiles, which can enhance both yield and quality, particularly in aromatic herbs such as basil. This highlights the potential of vertical farming for producing high‐value crops with consistent quality and for its contribution to more sustainable and resilient urban food production systems, reducing reliance on traditional agriculture and mitigating environmental impacts.

## Funding

The authors acknowledge support from the Scottish Government Rural and Environment Science and Analytical Services Division (RESAS; projects JHI‐B1‐6 and JHI‐B5‐1), Innovate UK (projects 10076759 and 10071841), and the Biotechnology and Biological Sciences Research Council (BB/Z51441X/1).

## Conflicts of Interest

The authors declare no conflicts of interest.

## Supporting information


**Table S1:** Plant height, dry weight %, NDVI, Greenness Index, TPC, FRAP and volatiles (sum of peak areas cm^−2^ leaf) measured in four basil varieties grown in a vertical farm and in a glasshouse. Values represent means ± SE (*n* = 3). Different letters indicate significant differences among all Environment (E) × Variety (V) treatment combinations for each parameter (split‐plot ANOVA, Fisher's protected LSD, *p* < 0.05).
**Table S2:** Fifty major volatile compounds detected by HS‐SPME‐GC‐MS and annotated in four basil varieties grown in a vertical farm and in a glasshouse. Values represent means of peak areas cm^−2^ leaf ± SE (*n* = 3). Different letters indicate significant differences among all Environment (E) × Variety (V) treatment combinations for each volatile compound (split‐plot ANOVA, Fisher's protected LSD, *p* < 0.05). The color scale ranges from green (low intensity) through yellow (intermediate intensity) to orange/red (high intensity). n.d., not detected; RT, retention time.
**Table S3:** Peak areas cm^−2^ leaf (PA cm^−2^ leaf) ± SE (*n* = 3) used to calculate percentage contribution of dominant volatile compounds and volatile classes to the total volatile peak area (ΣPA) per cm^2^ of leaf in four basil varieties grown in a vertical farm and a glasshouse (Table 2, main text). ΣPA cm^−2^ leaf is the sum of peak areas per cm^2^ of leaf of the 50 major volatile compounds. OM, oxygenated monoterpene; PP, phenylpropanoid; n.d., not detected.

## Data Availability

The data that support the findings of this study are available in the Supporting Information of this article.
